# Single-Cell Monitoring of Activated Innate Immune Signaling by a d2eGFP-Based Reporter Mimicking Time-Restricted Activation of *IFNB1* Expression

**DOI:** 10.3389/fcimb.2021.784762

**Published:** 2022-01-18

**Authors:** Emil Aagaard Thomsen, Sofie Andersen, Mikkel Haarslev Schröder Marqvorsen, Kristian Alsbjerg Skipper, Søren R. Paludan, Jacob Giehm Mikkelsen

**Affiliations:** Department of Biomedicine, Aarhus University, Aarhus, Denmark

**Keywords:** IFNB1 transcription, IFNB1 reporter, flow cytometry, innate immunity, single-cell

## Abstract

The innate immune system represents a balanced first line of defense against infection. Type I interferons (IFNs) are key regulators of the response to viral infections with an essential early wave of IFN-β expression, which is conditional, time-restricted, and stochastic in its nature. The possibility to precisely monitor individual cells with active *IFNB1* transcription during innate signaling requires a robust reporter system that mimics the endogenous IFN-β signal. Here, we present a reporter system based on expression of a destabilized version of eGFP (d2eGFP) from a stably integrated reporter cassette containing the *IFNB1* promoter and 3’-untranslated region, enabling both spatial and temporal detection of regulated *IFNB1* expression. Specifically, this reporter permits detection, quantification, and isolation of cells actively producing d2eGFP in a manner that fully mimics IFN-β production allowing tracking of *IFNB1* gene activation and repression in monocytic cells and keratinocytes. Using induced d2eGFP expression as a readout for activated immune signaling at the single-cell level, we demonstrate the application of the reporter for FACS-based selection of cells with genotypes supporting cGAS-STING signaling. Our studies provide a novel approach for monitoring on/off-switching of innate immune signaling and form the basis for investigating genotypes affecting immune regulation at the single-cell level.

## Introduction

The ability of cells to respond to external stimuli is hardwired to the transcriptional machinery. By adjusting the transcriptional output of specific genes, cells can change phenotype and characteristics to better suit the extracellular environment. The intrinsic ability of cells to recognize and respond to pathogens represents a hallmark of innate immunity, and innate immune sensing triggers orchestration of transcriptional regulation. A core component of the response to viral pathogens is signaling by type I interferons (IFNs) (Isaacs and Lindenmann, 1957), which include the IFN-α family of proteins encoded by thirteen different genes and IFN-β encoded by the *IFNB1* gene (Wang and Fish, 2019). Type I IFN genes encode cytokines, which elicit signaling through binding to the IFNAR1 and 2 receptor complex, hence initiating a signaling cascade resulting in massive induction of interferon-stimulated genes (ISGs) (Rusinova et al., 2013). Although innate immune activation is delicately balanced, irregularities may occur under certain circumstances. Hence, excessive activation of this system can lead to a continuous state of inflammation, which may cause autoimmune diseases such as rheumatoid arthritis and systemic lupus erythematosus (SLE) (Hall and Rosen, 2010), whereas deficiencies related to the type I IFN signaling may compromise immunity and cause immunodeficiencies (Andersen et al., 2015; Ogunjimi et al., 2017).

Depending on cell type and pathogen, IFN expression can be initiated by different routes, which all involve a predefined specific motif. Such motifs, pathogen-associated molecular patterns (PAMPs) and damage- associated molecular patterns (DAMPs), are recognized by pattern recognition receptors (PRRs), such as retinoic acid-inducible gene 1 (RIG-I)-like receptors (RLRs) and cyclic GMP-AMP synthase (cGAS), which facilitate signaling leading to expression of type I IFNs (Wang and Fish, 2019). Cellular detection of viruses is mediated by PRR-directed recognition of viral nucleic acids, which may contain unique modifications acting as PAMPs. Additionally, the aberrant localization of nucleic acids may serve as a danger signal, and cytosolic DNA can serve as a DAMP. Central to these processes are the RIG-I RNA-sensing and cGAS DNA-sensing pathways (Zevini et al., 2017), by which detection of both virus-derived cytosolic DNA and viral PAMP-containing RNA species leads to phosphorylation, dimerization, and activation of IRF-3 and IRF-7 (Honda et al., 2005). Simultaneously, sensing of cytosolic RNA or DNA results in phosphorylation of the inhibitory protein IκB, which leads to degradation of IκB, allowing nuclear factor kappa-light-chain-enhancer of activated B-cells (NF-κB) to translocate to the nucleus (Gilmore, 2006). Activation of IRF3/-7 and NF-κB is essential for activation of IFN-β production.

Transcription of the *IFNB1* gene is initiated by assembly of the core *IFNB1* enhanceosome upstream of the *IFNB1* transcription start site (TSS). Four positive regulatory domains (PRDs) termed PRDII, PRDI, PRDIII, and PRDIV are located in this region. Assembly starts with the binding of IRF-3 dimers to PRDIII and PRDI (Wathelet et al., 1998). NF-κB p50/p65 heterodimers then recognize and bind the motif contained in PRDII (Thanos and Maniatis, 1992), and ATF-2/c-Jun heterodimers finally bind to PRDIV (Falvo et al., 2000). Additionally, HMGA1 is recruited independently of the four PRDs, but in association with NF-κB and ATF-2/c-Jun. This interaction strengthens the binding of NF-κB and ATF-2/c-Jun to PRDII and PRDIV, respectively (Thanos and Maniatis, 1992). The enhanceosome now serves as a docking station for the histone acetyltransferase KAT2B, leading to acetylation of the adjacent nucleosomes. KAT2B is replaced by CBP (Agalioti et al., 2000), which is allocated to the enhanceosome in complex with an RNA polymerase II (Pol II) leading to initiation of transcription (Kim et al., 1998) and upregulation of IFN-β production. Interchromosomal interactions mediated through proteins such as ThPOK support sustained transcription (Nikopoulou et al., 2018), whereas subsequent down-tuning of the response is governed by RNA-destabilizing elements, including one located in the 3’-untranslated region (3’-UTR) and one 5’ to the *IFNB1* translation stop codon (Whittemore and Maniatis, 1990). Also, transcription is restricted by the protein TRIM33 (Ferri et al., 2015), and the long non-coding RNA LUCAT1 was recently reported as a negative regulator of *IFNB1* expression and ISG induction (Agarwal et al., 2020).

*IFNB1* expression is often utilized as a readout for innate immune activation during viral infections. In studies of cellular response pathways, it is essential therefore to model *IFNB1* expression to follow immune activation at a single-cell level. However, despite the detailed understanding of *IFNB1* gene expression, current models and reporter systems do not fully reflect *IFNB1* activation in a manner where reporter readouts mimic activation of the endogenous *IFNB1* locus. Hence, among previous reporter vectors used to assay innate immune activity by *IFNB1* activation, some have been based on activation of a gene cassette containing the interferon-sensitive responsive element and therefore cannot be expected to reflect the actual transcription of *IFNB1* (Patel et al., 2009; Uccellini and Garcia-Sastre, 2018). Other reporters contain a synthetic chimeric promoter composed of two copies of the core *IFNB1* promoter (-280 to +20 bp) placed immediately after each other (Leblanc et al., 1990; Lin et al., 2000; Li et al., 2005; Guo et al., 2012; Gentili et al., 2015) or a fragment (-125 to +55 bp) of the murine *IFNB1* promoter (Sato et al., 2000). Most of these reporters have been based on activation of a luciferase or catalase reporter gene placed under the control of the inducible promoter. These reporter vectors are often used in transient transfection studies resulting in a massive number of vector copies within each cell, leading to an amplified signal representing the transfected population of cells. Such reporters produce an amplified signal with a wide detection range, but do not allow distinction between cells in a population and do not support studying details of *IFNB1* control and regulation at a single-cell level. An *IFNB1*-IRES-YFP reporter mouse does exist (Scheu et al., 2008), but this design lacks the destabilizing element and has not been adapted for general use including high-throughput approaches.

Here, we describe a novel cell reporter system based on the expression of d2eGFP from a 1-kb *IFNB1* promoter fragment containing all four PRDs and the immediate upstream sequence. To allow expression of the reporter to decline quickly after activation, similarly to the expression of IFN-β, we used d2eGFP, a destabilized version of eGFP (Kitsera et al., 2007), as the reporter gene and included the *IFNB1* 3’ UTR in the expression cassette. By inserting the reporter in cells using a lentiviral approach, we demonstrate immune activation of the reporter in monocytic THP1- cells and HaCaT keratinocytes and establish the integrated reporter as an approach for resolving the induction and termination of *IFNB1* transcription at the single-cell level in any cell type of interest.

## Material and Methods

### Cell Lines

THP-1 cells were kept at densities 2×10^5^ to 1×10^6^ cells/mL in RPMI1640 (Lonza, Basel, Switzerland) with glutamine, supplemented with 10% FBS and 1% P/S. THP-1 cells were purchased from ATCC (TIB-202). HaCaT cells were cultured in DMEM (Lonza, Basel, Switzerland) supplemented with 10% FBS and 1% P/S; cells were split when confluent. Hacat cells were kindly supplied by Dr. U.B. Jensen Department of Biomedicine, Aarhus University. HEK293T were cultured in DMEM (Lonza, Basel, Switzerland) supplemented with 5% FBS and 1% P/S and were split when confluent. All cells were kept at 37°C, 5% CO_2_ and humidified air. HEK239T cells were purchased from ATCC (CRL-11268). Clonal expansion was performed in 96-well plates by seeding 0.5 cells per well. HaCaT clones were expanded using their regular growth medium, whereas THP-1 clones were expanded in medium supplemented with 30% FBS.

### Plasmid Construction

The reporter plasmid, pCCL/IFNB1-d2eGFP-3’UTR, was constructed by digesting pCCL/PGK-eGFP (Jakobsen et al., 2009) with BoxI and XhoI and inserting (a) a 1-kb fragment containing the *IFNB1* promoter region (upstream from the coding sequence) amplified from THP-1 genomic DNA, (b) the d2eGFP gene amplified from pT2/UAS-d2eGFP.SV40-Neo (Staunstrup et al., 2011), and (c) the *IFNB1* 3’-UTR amplified from THP-1 genomic DNA. The three fragments along with the digested backbone were assembled using NEBuilder^®^ HiFi DNA Assembly Master Mix. The resulting plasmid was confirmed by sanger sequencing.

### Lentiviral Vector Production

Lentiviral production was performed in HEK293T cells as previously described (Ryo et al., 2019). The multiplicity of Infection (MOI) used for transduction was estimated based on lentiviral vector preparations produced with pCCL/PGK-eGFP in parallel with the reporter vector.

### *IFNB1* Induction Assays and Reagents

Lipofectamine 2000 (ThermoFischer Scientific, Waltham, MA, USA) was used for transfection of dsDNA or Poly I:C. dsDNA (including Texas-red labeled dsDNA) was purchased as single-stranded oligos from TAG Copenhagen (Copenhagen, Denmark). Poly I:C and 2’3’-cGAMP were purchased from *In vivo*gen (San Diego, CA, USA). HSV-1 (KOS strain) was propagated as described previously (Bodda et al., 2020). In some cases, THP-1 cells were treated with phorbol 12-myristate 13-acetate (PMA). PMA (200 nM), PMA was added 48 hours before experiment start, and medium was changed to standard medium without PMA 24 hours later. Assays involving PMA-treated cells were performed in 24-well plates, with 2.5x10^5^ cells/well in 500 µL, cells were detached using 0.25% trypsin + EDTA. If not otherwise specified, *IFNB1* induction assays in THP-1 cells were performed in 96-well plates in a total of 60 µL medium, with 5×10^5^ cells/mL. HaCaT cells were seeded in 12-well plates with 5x10^5^ cells/well in 500 µL total volume. IFN-β secreted to the medium was quantified using HEK-Blue™ IFN-α/β cells and reagents from *In vivo*gen (San Diego, CA, USA).

### Flow Cytometry and Fluorescence Activated Cell Sorting (FACS)

The d2eGFP signal following treatment was quantified using flow cytometry. Samples were prepared in FACS buffer (PBS, 1% BSA, 2.5 mM EDTA and 25 mM HEPES). Propidium iodide (PI), final concentration 1.25 mg/mL, was included to identify and exclude dead cells. For experiments with Texas-red labeled dsDNA no PI were included. Fluorescence was quantified on a Novocyte 2100 (Agilent, Santa Clara, Ca, USA) Analyzer. For FACS analysis, cells were washed twice in PBS and re-suspended in FACS buffer with propidium iodide (PI), final concentration 1.25 mg/mL; cells were kept on ice. FACS was carried out on a 3 laser FACSAria III sorter (BD Biosciences, Franklin Lakes, New Jersey, USA). All instruments related to flow cytometry were serviced and maintained by the FACS Core Facility, Aarhus University. All flow data was analyzed using FlowJo version 10.0.7.

### RT-qPCR

For quantification of transgene cassette copy number, genomic DNA was extracted using a standard NaCl/EtOH precipitation protocol. The cassette was detected by quantifying copy numbers of the WPRE sequence present in the lentiviral vector, whereas the number of cells was estimated based on assessment of copy number of the albumin gene. Standard curves based on either pCCL/IFNB1-d2eGFP-3’UTR or pAlbumin [pAlbumin was a gift from Didier Trono (Addgene plasmid # 22037)] were used for absolute quantification and calculation of copy numbers of each element.

Total RNA was extracted by lysing cells with TRI Reagent™ Solution (Thermo Fisher). Phases were separated with chloroform, the aqueous phase mixed with absolute ethanol was transferred to columns from the High Pure miRNA Isolation Kit (Roche), and the columns were washed and eluted according to protocol. RNA purity and quantity was evaluated on a DeNovix DS-11 Spectrophotometer. 500 ng DNase-treated RNA was used for cDNA synthesis with Maxima H Minus RT (Thermo Scientific), samples were diluted 4 times, and qPCR was performed on a LightCycler 480 (Roche) with TaqMan Gene Expression Assays for *IFNB1* and *ACTB* (Thermo Fisher). Detection of d2eGFP was performed with a custom d2eGFP primer and probe set, sequences in **Supplementary File 2**.

### CRISPR-Based Knockout by Nucleofection

For nucleofection, 6 µg of recombinant spCas9 (Alt-R spCas9 nuclease V3, IDT, NJ, USA) and 3.2 µg of synthetic sgRNAs (Synthego, CA, USA) were mixed in 2 uL total and incubated 15 mins at 25°C to form Cas9/sgRNA RNP complexes. For each nucleofection experiment 3x10^5^ cells in 18 µL OptiMEM were added to the RNP complexes and electroporated on a Lonza 4D-Nucleofector™, with P3 solution settings and pulse code CM 138.

### Indel Detection

Genomic DNA was extracted using a standard NaCl/EtOH precipitation protocol. Following extraction, PCR was carried out, and the resulting amplicon was purified and subjected to Sanger sequencing. Indels were then quantified by ICE-analysis. Sequences for sgRNAs and primers are available in **Supplementary File 2**.

### Western Blot

THP1-IBER C3 and B6, were seeded in 12-well plates with 5x10^5^ cells per well and stimulated with 31.25 μg/mL cGAMP with or without Human IFNα 2a (PBL Assay Science).12 hours after treatment, cells were centrifuged and lysed in Pierce RIPA buffer (Thermo Scientific) supplemented with 10 nM NaF, complete protease inhibitor cocktail (Roche), and 5 U/mL benzonase (Sigma) at 80,000 cells/100 uL. 14 uL cell lysate was run on a 4-15% SDS polyacrylamide gel (BIO-RAD) and transferred to a PVDF membrane (BIO-RAD). The membrane was cut according to analyte molecular masses and stained for vinculin (hVIN-1, Sigma), MX1 (D3W71, Cell Signaling), IFIT1 (D2X9Z, Cell Signaling), or ISG15 (22D2, Cell Signaling) as appropriate. The primary antibodies were followed by either HRP-conjugated anti-mouse antibody (Jackson Immuno research) for the anti-vinculin antibody or HRP-conjugated anti-rabbit (Jackson Immuno research) for the anti-MX1, IFIT1, and ISG15 antibodies. The Western blot was developed using SuperSignal West Dura substrate (Thermo Scientific) for vinculin blots or SuperSignal West Femto substrate (Thermo Scientific) for MX1, IFIT1, and ISG15 and visualized on a ChemiDoc Imager (BIO-RAD).

## Results and Discussion

### Generation of a d2eGFP-Based *IFNB1* Reporter

**Figure 1:**
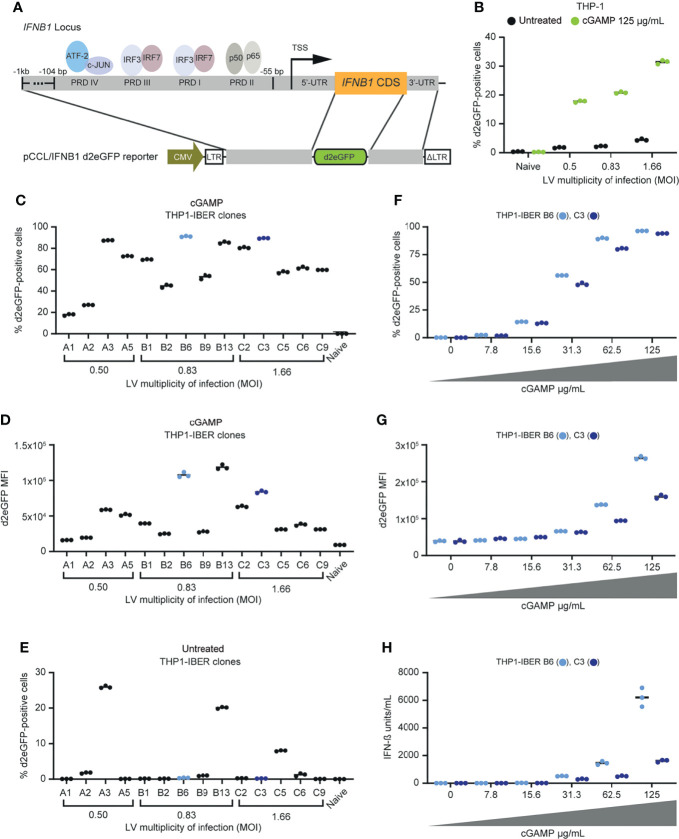
Generation of stable monogenic IFN-β reporter (IBER) clones. **(A)** Schematic representation of the *IFNB1* locus and the genomic regions of this locus incorporated into pCCL/IFNB1-d2eGFP-3’UTR. **(B)** Initial validation of successful transduction with LV/IFNB1-d2eGFP-3’UTR by treatment with cGAMP (125 µg/mL) for 12 hours d2eGFP-positive cells were measured by flow cytometry. **(C)** Screening of 14 different THP1-IBER clones with cGAMP (62.5 µg/mL) for 12 hours and subsequent detection of the percentage d2eGFP-positive cells by flow cytometry. **(D)** Percentage of d2eGFP-positive cells in untreated THP1-IBER clones. **(E)** Quantification of d2eGFP MFI of each THP1-IBER clone following cGAMP treatment. **(F)** Percentage d2eGFP-positive cells after treatment of the two THP1-IBER clones B6 and C3 with different dosages of cGAMP (0-125 µg/mL) for 12 hours. **(G)** d2eGFP MFI of clones B6 and C3 after cGAMP treatment. **(H)** Medium from the same cells was collected before flow cytometry, and the concentration of IFN-β (units/mL) was quantified by HEK-Blue™ IFN-α/β. Experiments were performed in biological triplicates (individual wells); each panel represents one experiment.

To mimic expression of IFN-β from the endogenous *IFNB1* locus, we designed a reporter mimicking the genomic context of *IFNB1* with the coding sequence (CDS) of *IFNB1* replaced by the d2eGFP reporter gene encoding a destabilized version of the enhanced green fluorescent protein with a rapid turn-over (Kitsera et al., 2007). Expression of d2eGFP was driven by a short 1-kb long sequence containing the promoter segment, including the core *IFNB1* enhanceosome and the 5’-UTR, derived from the endogenous *IFNB1* promoter (**Figure 1A**). The d2eGFP gene was flanked downstream by the *IFNB1* 3’-UTR allowing for regulation of the d2eGFP-encoding transcript by RNA-destabilizing elements and recognition by microRNAs. The reporter cassette was inserted into a lentiviral vector (plasmid referred to as pCCL/IFNB1-d2eGFP-3’UTR) to allow easy genomic insertion in cells of interest, including hard-to-transfect cells.

Following transduction of monocytic THP-1 cells with the lentiviral vector containing the reporter at different MOIs, we treated the cells with cyclic guanosine monophosphate-adenosine monophosphate (cGAMP) resulting in a clear induction of d2eGFP 12 hours after treatment (**Supplementary Figure S1A**). We also observed a marked cGAMP-induced increase in d2eGFP-positive cells with increasing MOI and noted a limited background d2eGFP signal in cells that were not treated with cGAMP (**Figure 1B**). Moreover, d2eGFP expression in the cell pool was activated by transfection with dsDNA resulting in between 10-13% d2eGFP-positive cells depending on the MOI, whereas poly I:C only induced d2eGFP expression to a limited extent (**Supplementary Figure S2**), which is in line with the low expression of RIG-I in THP-1 cells (Imaizumi et al., 2007).

We then established cell clones harboring the IFNB1-d2eGFP-3’UTR cassette. We refer to these cells as *IFNB1* Expression Reporter (IBER) cells. Among a panel of THP1-IBER clones, we selected 14 clones in which d2eGFP expression was activated upon stimulation with cGAMP. Following cGAMP treatment, we identified d2eGFP expression profiles indicating considerable interclonal variation ranging from 18 ± 0.6% d2eGFP-positive cells in THP1-IBER clone A1 to 91 ± 0.3% in THP1-IBER clone B6 (**Figure 1C**). Also, d2eGFP intensities varied substantially between the THP1-IBER clones (**Figure 1D**), whereas only a weak correlation between the fraction of d2eGFP-positive cells and the d2eGFP median fluorescence intensity (MFI) (R^2^ = 0.66) was observed (**Supplementary Figure S3A**). Moreover, we did not observe a direct correlation between vector copy numbers measured by qPCR (**Supplementary Figure S3B**) and d2eGFP expression levels after cGAMP treatment (MFI) (R^2^ = 0.22) (**Supplementary Figure S3C**). Although most THP1-IBER clones exhibited little or no d2eGFP expression in the absence of cGAMP, at least three THP1-IBER clones (A3, B13, and C5) showed considerable background expression (**Figure 1E**). Most likely, the background signal in some clones indicated that expression of the integrated d2eGFP cassette in these clones was affected by the chromosomal environment and potentially influenced by a nearby constitutively active endogenous promoter. Altogether, these findings indicated that the level of d2eGFP expression was affected by basic clonal variation and correlated only partially with the number of vector insertions in each clone. Additionally, we verified that signaling by type I IFNs through the IFNAR1/2 receptor did not directly trigger d2eGFP expression, demonstrating that type I IFN signaling did not contribute to background activation of the integrated d2eGFP cassette (**Supplementary Figure S4**).

### Characterization of THP1-IBER Clones

Two THP1-IBER clones, B6 and C3, showed solid d2eGFP induction upon treatment with cGAMP (high percentage of d2eGFP-expressing cells with high MFI) with no background in the absence of cGAMP (**Figure 1**), and were selected for further studies. For both THP1-IBER clones, treatment with cGAMP without prior treatment with PMA produced a dose-dependent d2eGFP signal measured 12 hours after treatment (**Figures 1F, G**). The response was strongest in THP1-IBER clone B6, leading to both a higher fraction of d2eGFP-positive cells and a higher d2eGFP intensity. By functionally assessing the IFN-β units secreted into the culture medium, a similar dose-dependent induction of IFN-β (**Figure 1H**) was observed for both THP1-IBER clones (**Figure 1H**), indicating that the d2eGFP induction pattern mimicked cGAMP-induced IFN-β production from the endogenous *IFNB1* alleles and that variation between the two THP1-IBER clones reflected clonal differences in the capacity to respond to cGAMP rather than vector copy numbers.

**Figure 2:**
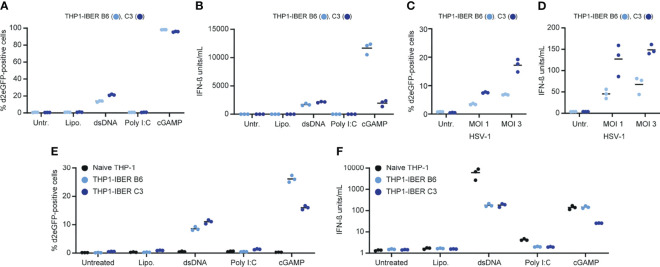
Characterization of the *IFNB1* d2eGFP reporter. **(A)** Treatment of THP1-IBER clone B6 and C3 with lipofectamine (4 µL/mL), dsDNA (4µg/mL), Poly I:C (4µg/mL) or cGAMP (125 µg/mL) and subsequent detection of percentage d2eGFP-positive cells 12 hours after treatment measured by flow cytometry. **(B)** Medium harvested prior to flow cytometry, and the IFN-β (units/mL) was detected by HEK-Blue™ IFN-α/β. **(C)** Treatment of PMA differentiated THP1-IBER clones with HSV-1[KOS] at different MOI (1 or 3). **(D)** Quantification of IFN-β secreted to the media following treatment with HSV-1[KOS]. **(E)** Treatment of PMA-differentiated naïve THP-1 cells or THP1-IBER clone B6 and C3 with lipofectamine (4 µL/mL), dsDNA (4µg/mL), Poly I:C (4µg/mL) or cGAMP (125 µg/mL). The fraction of d2eGFP positive cells was determined by flow cytometry 12 hours after treatment. **(F)** In medium harvested prior to flow cytometry, the level of IFN-β (units/mL) was quantified by HEK-Blue™ IFN-α/β. Experiments were performed in biological triplicates (individual wells); each panel represents one experiment.

Next, we verified the ability of the THP1-IBER clones to respond to different stimuli. Again, cGAMP induced strong d2eGFP activation reflecting the direct activation of STING, and the reporter was also turned on by transfection of dsDNA into the cells, indicative of dsDNA recognition by cGAS and subsequent activation of STING (**Figure 2A**). Notably, whereas the d2eGFP signal intensity was higher in THP1-IBER B6 than in C3, the response to dsDNA was strongest in C3 (**Figure 2A** and **Supplementary Figure S5A**). The same pattern was evident for the amounts of secreted IFN-β (**Figure 2B**). Due to the differences in the magnitude of the responses to cGAMP and dsDNA, we treated three additional THP1-IBER clones (C2, C9, and B1) with the three stimuli and observed similar d2eGFP expression patterns (**Supplementary Figures S5B, C**).

The THP1-IBER clones B6 and C3 were differentiated into macrophage-like cells with PMA. The clones were then exposed to herpes simplex virus type 1 (HSV-1), and d2eGFP expression was assayed by flow cytometry 10 hours later. Whereas the untreated samples showed no induction of d2eGFP, expression was induced by infection by HSV-1 in an MOI-dependent fashion in the reporter cells, leading to most prominent activation in clone C3 (**Figure 2C**). Also in this case, d2eGFP induction in the two cell lines matched IFN-β secretion (**Figure 2D**). Finally, we subjected PMA-differentiated reporter cells to dsDNA, Poly I:C, and cGAMP and examined d2eGFP and IFN-β induction levels. Again, under these conditions, the reporter mimicked the endogenous *IFNB1* response (**Figures 2E, F**), although *IFNB1* expression levels after dsDNA treatment were markedly lower in the reporter THP1-IBER clones relative to naïve cells. This could indicate coincidental clonal variation or that induction of expression from the endogenous *IFNB1* locus was affected by the presence of the reporter cassette.

### Induction of the d2eGFP Reporter by Poly I:C in HaCaT Cells

**Figure 3:**
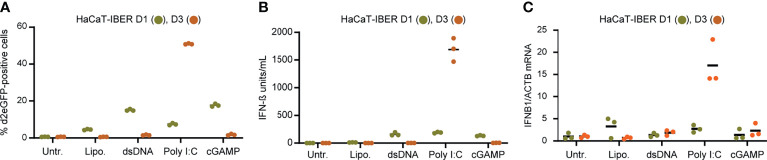
*IFNB1* d2eGFP reporter in HaCaT cells. **(A)** HaCaT-IBER clones D1 and D3 were treated with lipofectamine (4 µL/mL), dsDNA (4µg/mL), Poly I:C (4µg/mL) or cGAMP (125 µg/mL), and the fraction of d2eGFP positive cells was determined by flow cytometry 12 hours after treatment. **(B)** Quantification of IFN-β (units/mL) in the medium by HEK-Blue™ IFN-α/β. **(C)** RNA was extracted from cells and mRNA levels of *IFNB1* and *ACTB* were quantified by qPCR. Experiments were performed in biological triplicates (individual wells); each panel represents one experiment. For qPCR, each biological replicate was analyzed in technical duplicates.

To examine the capacity of the reporter construct to mimic activation of innate immune signaling induced by RNA sensing, we transduced HaCaT keratinocytes with LV/IFNB1-d2eGFP-3’UTR. The resulting HaCaT-IBER cell population was then treated with dsDNA, Poly I:C, or cGAMP, which in all cases resulted in activation. Among cells treated with Poly I:C, 16.4 ± 1.7% of the cells were positive for d2eGFP (**Supplementary Figure S6**). Notably, two HaCaT-IBER clones, D1 and D3, expanded from this cell population, responded quite differently to stimuli. Whereas D1 showed a moderate d2eGFP response to dsDNA, Poly I:C, and cGAMP, the response to Poly I:C was pronounced in D3 with more than 50% of the cells expressing d2eGFP (**Figure 3A**). D3, on the other hand, did not express d2eGFP upon stimulation with dsDNA or cGAMP. This difference was evident also for *IFNB1* expression based on levels of both protein (**Figure 3B**) and mRNA levels (**Figure 3C**), suggesting that clonal variation had marked influence on the innate signaling profile.

### Spatial and Temporal Detection of IFN-β Expression by d2eGFP Reporter Signal

**Figure 4:**
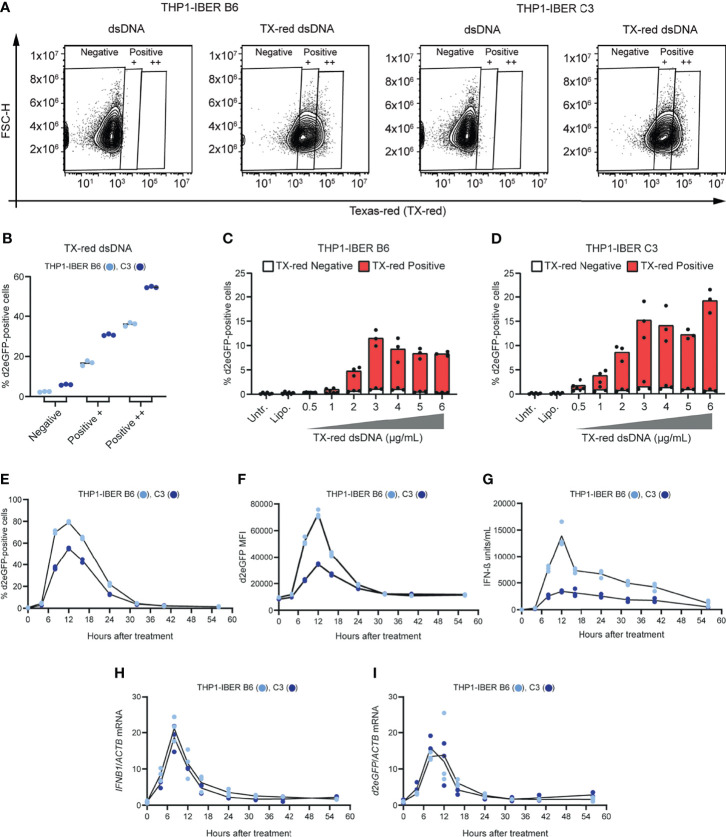
Stochastic behaviour of the *IFNB1* d2eGFP reporter and temporal monitoring. **(A)** Treatment of THP1-IBER clones B6 and C3 with 4 (µg/mL) dsDNA or TX-dsDNA. The fraction Texas-red positive cells was determined by flow cytometry 12 hours after treatment. **(B)** The fraction of d2eGFP-positive cells from each on the Texas-red signal resulting in gates: Negative, Positive +, and Positive ++. **(C)** TX-dsDNA dose response with 0 to 6 µg/mL. The fractions of d2eGFP-positive cells and Texas-red positive cells were determined by flow cytometry 12 hours after treatment in THP1-IBER clone B6 and **(D)** clone C3. **(E)** THP1-IBER clones B6 and C3 were treated with cGAMP (31.25 µg/mL), and flow cytometry was used to quantify the fraction of d2eGFP-positive cells over time, from 0-56 hours. **(F)** The magnitude of the response as reflected by the d2eGFP MFI. **(G)** Medium was used to quantify secreted IFN-β (units/mL) at the different time points by HEK-Blue™ IFN-α/β. **(H)** RNA was extracted from cell pellets harvested at each time point, and mRNA levels of *IFNB1* and *ACTB* were quantified by qPCR. **(I)** Quantification of d2eGFP and ACTB mRNA levels by qPCR. 5x10^5^ cells/mL were seeded in 500 µL total in 12-well plates. Experiments were performed in biological triplicates (individual wells); each panel represents one experiment. For qPCR, each biological replicate was analyzed in technical duplicates.

Variation in IFN-β production between cells is affected by the stochastic nature of *IFNB1* transcription (Ford and Thanos, 2010). As opposed to assays based on mRNA expression or activation of a luciferase reporter, a d2eGFP-based reporter allows detection of activated signaling at the single-cell level. To demonstrate the presence of such random variation, the THP1-IBER clones were transfected with dsDNA-labeled with Texas-red, enabling us to assay for activation of *IFNB1* expression (green cells) within the fraction of cells that was indeed exposed to foreign dsDNA in the cytoplasm (red cells). We first verified that the reporter response to Texas-red dsDNA (TX-dsDNA) was comparable to the response to standard dsDNA (**Supplementary Figure S7A**) and then plotted the fraction of d2eGFP-positive cells within each of the gates defined as ‘negative’, ‘positive +’ and ‘positive ++’ in the channel detecting Texas-red using the dsDNA samples to set the gates (**Figures 4A, B**). The negative gate contained 2.4 ± 0.2% (B6) and 5.9 ± 0.2% (C3) d2eGFP-positive cells. For THP1-IBER clones B6 and C3 ‘positive +’ cells showed 16.7 ± 0.9% and 30.7 ± 0.4% d2eGFP-positive cells, respectively, whereas the corresponding percentages for ‘positive ++’ cells increased to 35 ± 0.8% and 57 ± 0.3%. We observed a dose-dependent increase of both Texas-red- and d2eGFP-positive positive cells (**Supplementary Figures S7B, C**), which was saturated at 3 μg/mL and higher concentrations. For both THP1-IBER clones, almost all d2eGFP-positive cells were positive for Texas-red, whereas all Texas-red-negative cells were negative for d2eGFP (**Figures 4C, D**). Together, these data showed that the THP1-IBER clones responded to dsDNA in a dose-dependent manner. Furthermore, similar to previous studies using *in-situ* hybridization (Zawatzky et al., 1985; Enoch et al., 1986) and single-cell sorting coupled with qPCR (Hu et al., 2007), the IBER configuration unveiled the subset of exposed cells responsible for the IFN-β production.

To investigate whether induction, shutdown, and turnover of the d2eGFP signal mimicked the *IFNB1* mRNA expression profile over time, we set out to track the d2eGFP signal in the two THP1-IBER clones B6 and C3 by flow cytometry. Notably, upon stimulation with cGAMP, a strong increase in the fraction of high-intensity d2eGFP-positive cells was detected (**Figures 4E, F**). For both clones, the signal peaked after 12 hours and subsequently declined to near-background levels 32 hours after stimulation. Moreover, the d2eGFP expression profile mimicked the *IFNB1* expression profile, measured by an IFN-β secretion assay (**Figure 4G**). Additionally, quantification of *IFNB1* mRNA (**Figure 4H**) and d2eGFP mRNA (**Figure 4I**) confirmed matching transcriptional profiles. Notably, *IFNB1* and d2eGFP mRNA levels peaked 8 hours after stimulation (**Figures 4H, I**), whereas both IFN-β and d2eGFP protein levels peaked 4 hours later (**Figures 4G, E**). Interestingly, both *IFNB1* and d2eGFP mRNA levels reached background levels 24 to 32 hours after stimulation, which was in accordance with the d2eGFP protein levels. In summary, these findings demonstrated that both stimulation and deactivation of the d2eGFP-based *IFNB1* reporter closely resembled the activity of the endogenous *IFNB1* locus upon stimulation of innate responses, providing a unique approach for modelling and evaluating innate signaling at the single-cell level over time. As expected, the impact on cell viability following cGAMP treatment increased over time. Hence, the percentage of living cells dropped to 41.9 ± 4.0% and 67.9 ± 1.0% for THP1-IBER B6 and C3, respectively 56 hours following treatment (**Supplementary Figure S7D**).

### Reporter-Based Identification of Cells With Functional and Dysfunctional Immune Signaling

**Figure 5:**
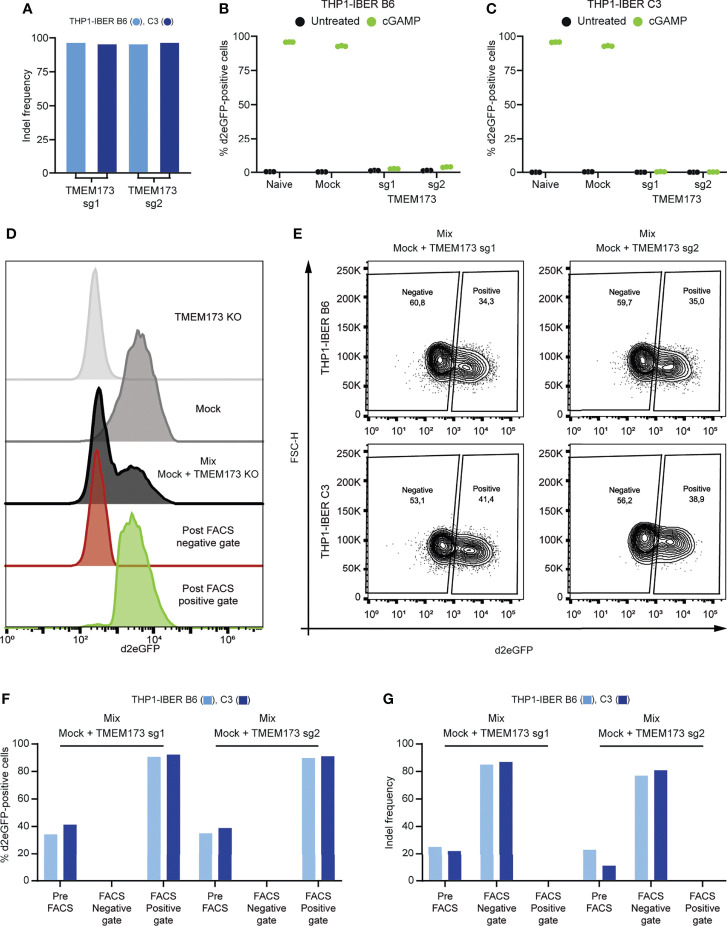
STING-pathway dependency and spatial resolved detection of d2eGFP. **(A)** Indel frequency determined by sanger sequencing and ICE-analysis following nucleofection with Cas9/sgRNA complexes. **(B)** Treatment with cGAMP in naïve, mock-nucleofected cells and *TMEM173* KO cells followed by detection of d2eGFP-positive cells 12 hours after treatment by THP1-IBER clone B6 and **(C)** clone C3 by flow cytometry. **(D)** Histograms of different cGAMP-treated samples before and after FACS. **(E)** Mixed populations of mock and *TMEM173* KO populations in both clones, treated with cGAMP for 12 hours and subjected to FACS. **(F)** Detection of d2eGFP-signal in different populations from both THP1-IBER clones before and after FACS. **(G)** Detection of indel frequencies in different populations from both THP1-IBER clones before and after FACS. Experiments **(B, C)** were performed in biological triplicates (individual wells); each of the panels represents one experiment. FACS was performed on a single sample.

DNA sensing by the cGAS/STING pathway is essential for ignition of innate immune signaling by dsDNA and cGAMP (Paludan et al., 2019). To examine whether d2eGFP activation by cGAMP in THP1-IBER cells was dependent on STING, we knocked out the *TMEM173* gene encoding STING using two different sgRNAs in both THP1-IBER clones B6 and C3 (**Figure 5A**) and treated *TMEM173* knockout and control cells with cGAMP. d2eGFP levels, measured after 12 hours, showed strong induction of d2eGFP production in control cells (above 90% d2eGFP cells), whereas expression of d2eGFP was very low or absent in *TMEM173* knockout cells (**Figures 5B, C**), thus confirming that activation of the d2eGFP reporter was dependent on STING. Based on this finding, we wanted to demonstrate the use of the d2eGFP reporter to distinguish cells with functional innate signaling from cells with a defective signaling response. For this purpose, we verified that cGAMP-treated reporter cells could be effectively separated from cGAMP-treated *TMEM173* knockout cells by fluorescent activated cell sorting (FACS) (**Figure 5D** and **Supplementary Figure S8**). Next, for both reporter THP1-IBER clones and for both *TMEM173*-targeting sgRNAs, a mix of *TMEM173* knockout cells and ‘mock’ cells that did not receive sgRNAs was treated with cGAMP, allowing subsequent FACS-based separation of d2eGFP-positive from d2eGFP-negative cells (**Figure 5E**). For both clones and both sgRNAs, d2eGFP-positive cells were clearly separated from the remaining cell population (**Figure 5F**). We then determined the indel rates in the *TMEM173* locus in all FACS-generated cell populations. Based on the d2eGFP signal, the population was separated in two, a d2eGFP-negative population with knockout of STING production (indel rates of 80%) and a d2eGFP-positive population with functional signaling and no evidence of indels in *TMEM173* (**Figure 5G**). Collectively, these findings demonstrated the use of the IBER reporter cells to select for specific genotypes affecting innate immune signaling based on the activation, or alternatively deactivation, of d2eGFP production mimicking the *IFNB1* expression profile at the single-cell level.

Our work demonstrates the potential of a novel approach for monitoring activated signaling through the cGAS-STING pathway in monocytes and keratinocytes. Using production of d2eGFP as the primary readout for stimulated signaling, we generate a reporter that fully mimics regulation of expression of IFN-β from the endogenous *IFNB1* locus, allowing easy FACS-based separation of actively signaling cells from cells that are not signaling. Such reporter cells make it possible to follow immune activation and deactivation at the single-cell level allowing high-throughput experiments and library approaches to be carried out. Relative to existing reporter systems, our reporter provides substantial benefits including lentiviral delivery, which enables use in any cell line that is permissible for transduction, including hard-to-transfect cells. Also, with an expected half-life of d2eGFP of ~5.5 hours, compared to 26 hours for wild type eGFP (Kitsera et al., 2007) studies focusing on the termination of innate immune signaling are feasible allowing shutdown of the response to be tracked in finer detail. Besides regular flow cytometry, the IBER system is compatible with FACS permitting the separation of single cells. FACS does not only allow for the separation of genotypes, as demonstrated here, but also separation of cells responding differently to treatments affecting activation of innate immunity. Here, we provide THP-1 and HaCaT cell lines that are immediately suited for studies of immune signaling. It should be noted, however, that use of the reporter vector in other cell types will require confirmation of the expected activation profile. Hence, it cannot be excluded that the specific integration site may in some cases interfere with transcription of d2eGFP, potentially providing a d2eGFP profile signal that differs from the *IFNB1* transcription profile. Also, an inserted reporter cassette may not always reflect regulatory mechanisms linked to the chromatin environment surrounding the endogenous *IFNB1* gene entirely. Collectively, our studies provide a new and powerful tool for detection of innate immune activation as well as reporter cell lines available for immediate use for monitoring active immune signaling at the single-cell level.

## Data Availability Statement

The original contributions presented in the study are included in the article/**Supplementary Material**. Further inquiries can be directed to the corresponding author.

## Author Contributions

ET and JM conceived the project and designed the experiments. ET, SA, KS, and MM performed the experiments. SP and JM supervised the findings of this work, and ET and JM wrote the manuscript and assembled the figures. All authors contributed to the article and approved the submitted version.

## Funding

This work was made possible through support from the Novo Nordisk Foundation (NNF17OC0029042) and The European Research Council (ERC-AdG ENVISION; 786602). ET is funded by a PhD fellowship from the Graduate School of Health, Aarhus University.

## Conflict of Interest

The authors declare that the research was conducted in the absence of any commercial or financial relationships that could be construed as a potential conflict of interest.

## Publisher’s Note

All claims expressed in this article are solely those of the authors and do not necessarily represent those of their affiliated organizations, or those of the publisher, the editors and the reviewers. Any product that may be evaluated in this article, or claim that may be made by its manufacturer, is not guaranteed or endorsed by the publisher.

## Supplementary Material

Supplementary File 1 Pdf file containing all supplementary figures.

Supplementary File 2 Excel spreadsheet containing data used for figures and sequences of primers. All generated FCS files can be accessed through: https://flowrepository.org/id/RvFrlqyhtH4W9vObwKwWEu76G6my896Y45Xgxj9vTJwb61qOol0AzrGxRpvNlsaQ. Plasmid will be deposited at addgene.org
