# Supplementary material for: Single-Cell Monitoring of Activated Innate Immune Signaling by a d2eGFP-Based Reporter Mimicking Time-Restricted Activation of IFNB1 Expression
Source: Front Cell Infect Microbiol. 2022 Jan 18;11:784762. doi: 10.3389/fcimb.2021.784762 (PMC8803904; doi:10.3389/fcimb.2021.784762)
Supplement: Supplementary File 1 — Pdf file containing all supplementary figures. [file DataSheet_1.pdf]

Supplementary Figure S1

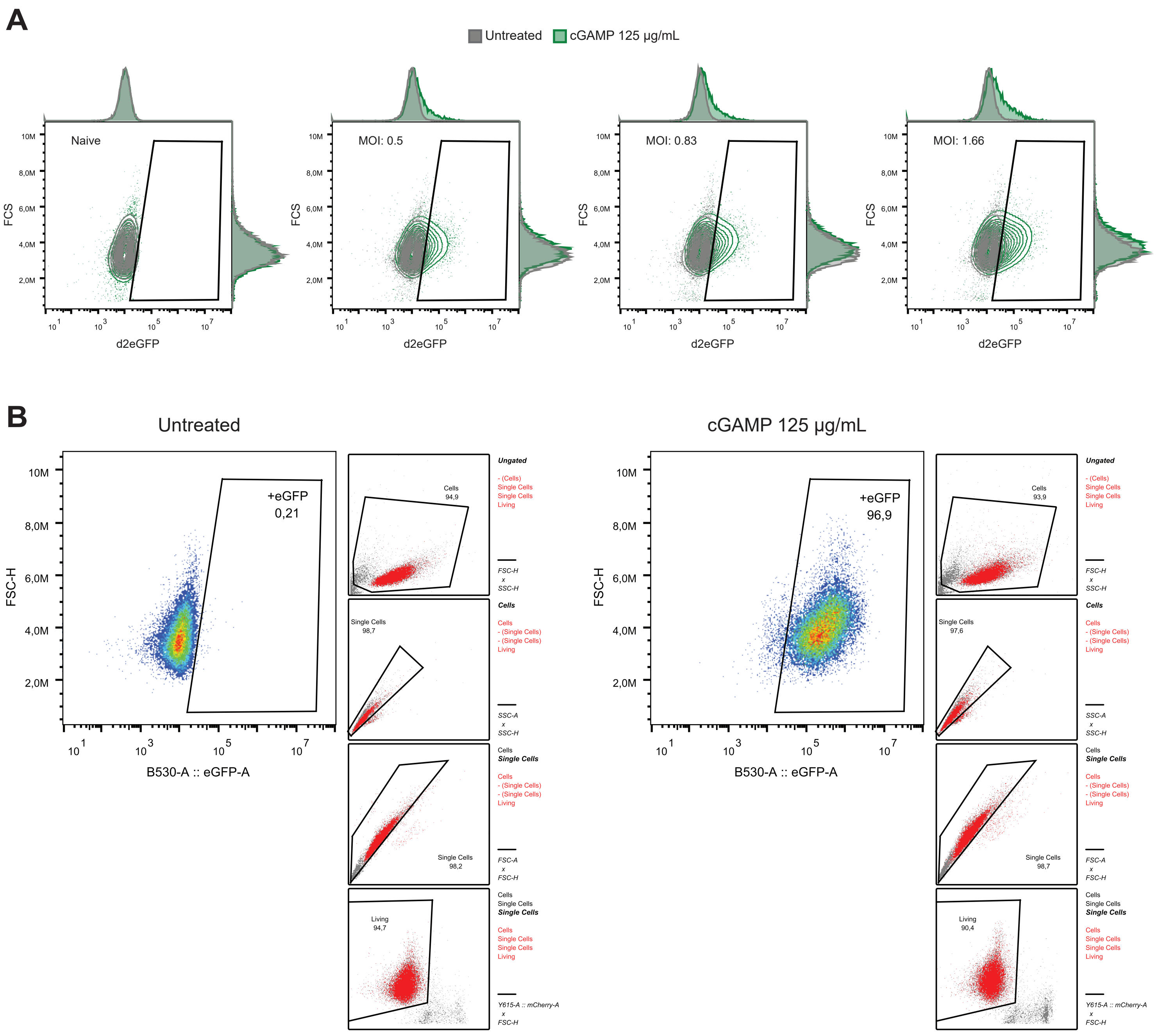

Supplementary Figure S1. (A) Histogram overlays of untreated and cGAMP-treated (125 µg/mL) THP1-IBER populations. **(B)** Gating strategy used to quantify d2eGFP signal in THP1-IBER cells.

Supplementary Figure S2

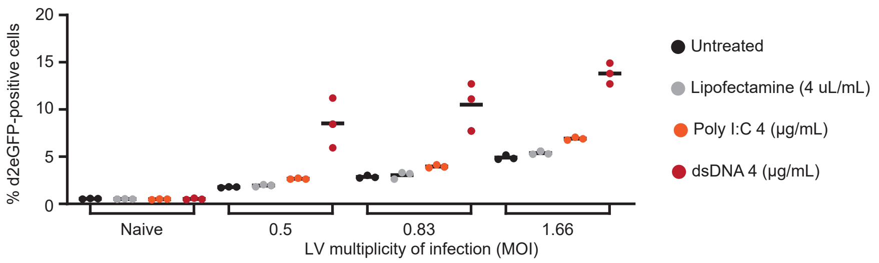

Supplementary Figure S2. Treatment of THP1-IBER pools with Lipofectamine (4 µL/mL), dsDNA (4 µg/mL), and Poly I:C (4 µg/mL); quantification of d2eGFP signal 12 hours after treatment. Experiments were performed in biological triplicates (individual wells); each panel represents one experiment.

Supplementary Figure S3

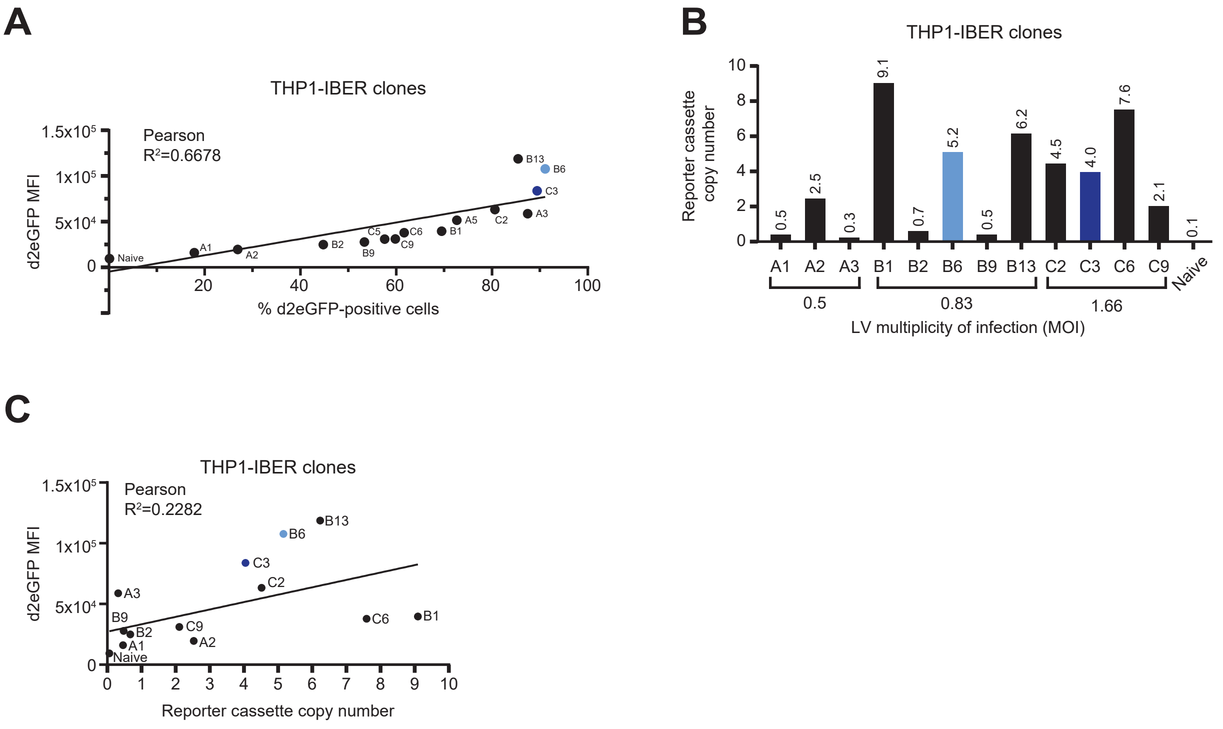

Supplementary Figure S3. (A) Values of d2eGFP MFI and percentage of positive cells for each THP1-IBER clone, plotted against each other and analysis of pearson correlation R^2=0.66. **(B)** Quantification of IFNB1 d2eGFP reporter cassette copy number by qPCR. Detection of the cassette was carried out using WPRE-specific primers, and albumin was used to assess the number of cells. **(C)** d2eGFP MFI plotted against qPCR-determined cassette copy number for each THP1-IBER clone and analysis of pearson correlation R^2=0.22. Experiments were performed in biological triplicates (individual wells); each panel represents one experiment. For qPCR, each biological replicate was analyzed in technical duplicates.

Supplementary Figure S4

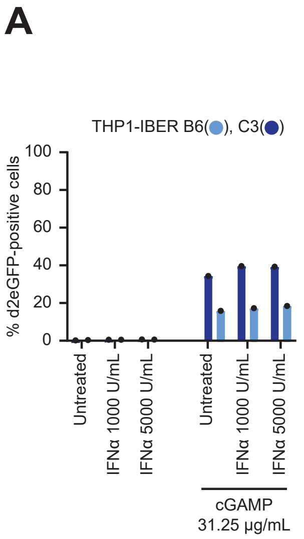

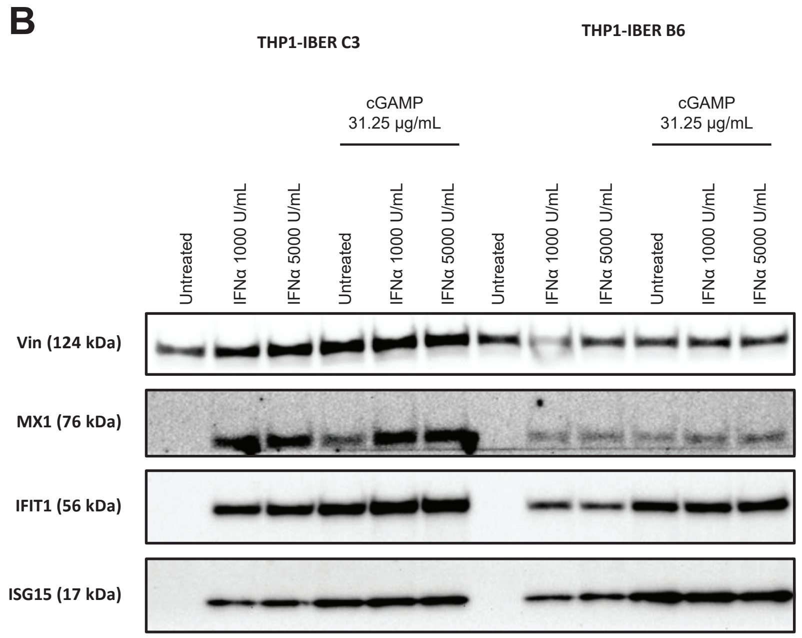

Supplementary Figure S4. (A) THP1-IBER clones B6 and C3 were treated with different doses of IFNα alone or in combination with cGAMP (31.25 μg/mL). The percentages of d2eGFP positive cells were detected by flow cytometry 12 hours after treatment. **(B)** Western blot of the ISGs MX1, IFIT1 and ISG15 to confirm IFNα signalling activity through IFNAR1/2. Experiments were performed twice in singlets, one is shown.

Supplementary Figure S5

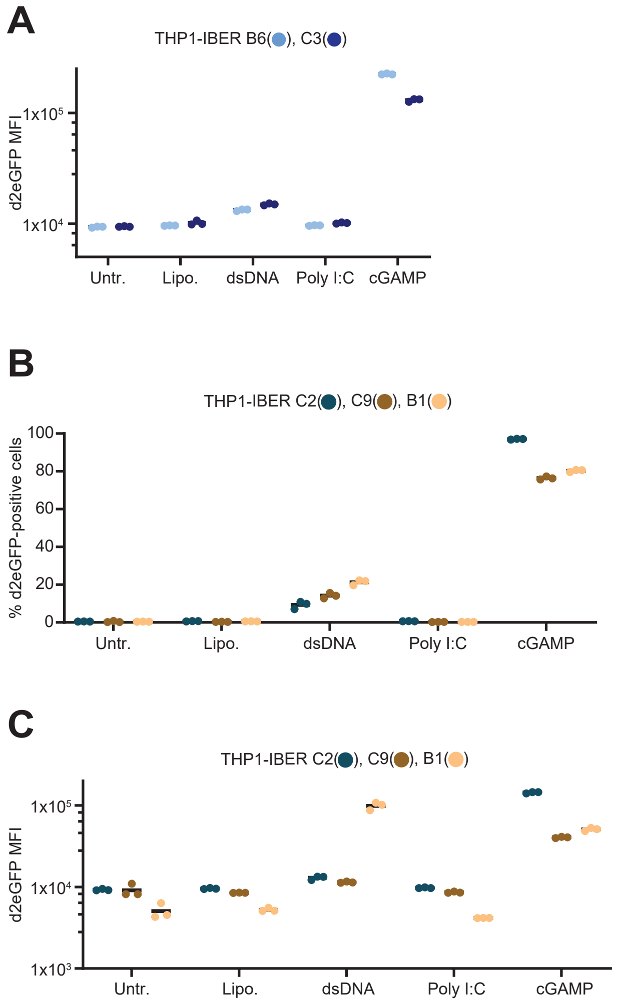

Supplementary Figure S5. (A) d2eGFP MFI values from THP1-IBER clones B-6 and C-3 treated with Lipofectamine (4 µL /mL), dsDNA 4 (µg/mL), Poly I:C (4 µg/mL), and cGAMP (125 µg/mL) for 12 hours. **(B)** Fraction of d2eGFP-positive cells in three additional THP1-IBER clones, C-2, C-9 and B-1, treated with lipofectamine (4 µL /mL), dsDNA (4 µg/mL), Poly I:C (4 µg/mL), and cGAMP (125 µg/mL) for 12 hours. **(C)** d2eGFP MFI of the THP1-IBER clones C-2, C-9 and B-1 12 hours after treatment. Experiments were performed in biological triplicates (individual wells); each panel represents one experiment.

Supplementary Figure S6

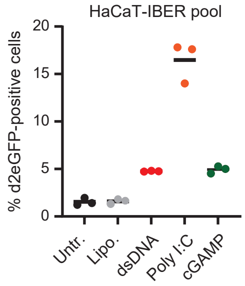

Supplementary Figure S6. The HaCaT reporter pool was treated with lipofectamine (4 μL /mL), dsDNA (4μg/mL), Poly I:C (4μg/mL) or cGAMP (125 μg/mL), and the fraction of d2eGFP-positive cells was determined by flow cytometry 12 hours after treatment. Experiments were performed in biological triplicates (individual wells); each panel represents one experiment.

Supplementary Figure S7

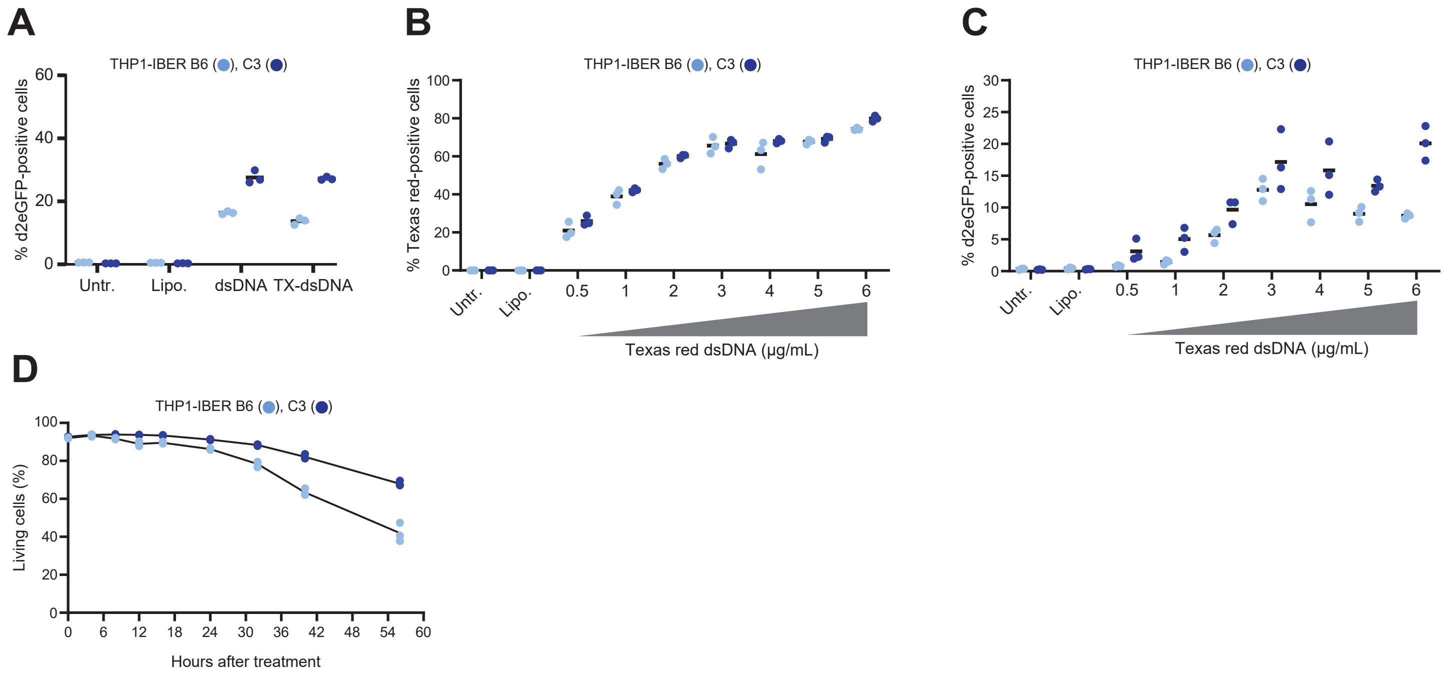

Supplementary Figure S7. (A) Treatment of THP1-IBER clones B-6 and C-3 with dsDNA (4 µg/mL) and TX-dsDNA (4 µg/mL); d2eGFP was quantified by flow cytometry 12 hours after treatment. **(B)** THP1-IBER clones B-6 and C-3 treated with a dose response of TX-dsDNA from 0 to 6 µg/mL; Texas-red was quantified by flow cytometry 12 hours after treatment. **(C)** Plotting of d2eGFP signal obtained by the dose response of TX-dsDNA. **(D)** THP1-IBER clones B6 and C3 were treated with cGAMP (31.25 µg/mL), and flow cytometry was used to quantify the fraction of living cells over time, from 0-56 hours. Living and dead cells were separated by PI-staining. Experiments were performed in biological triplicates (individual wells); each panel represents one experiment.

Supplementary Figure S8

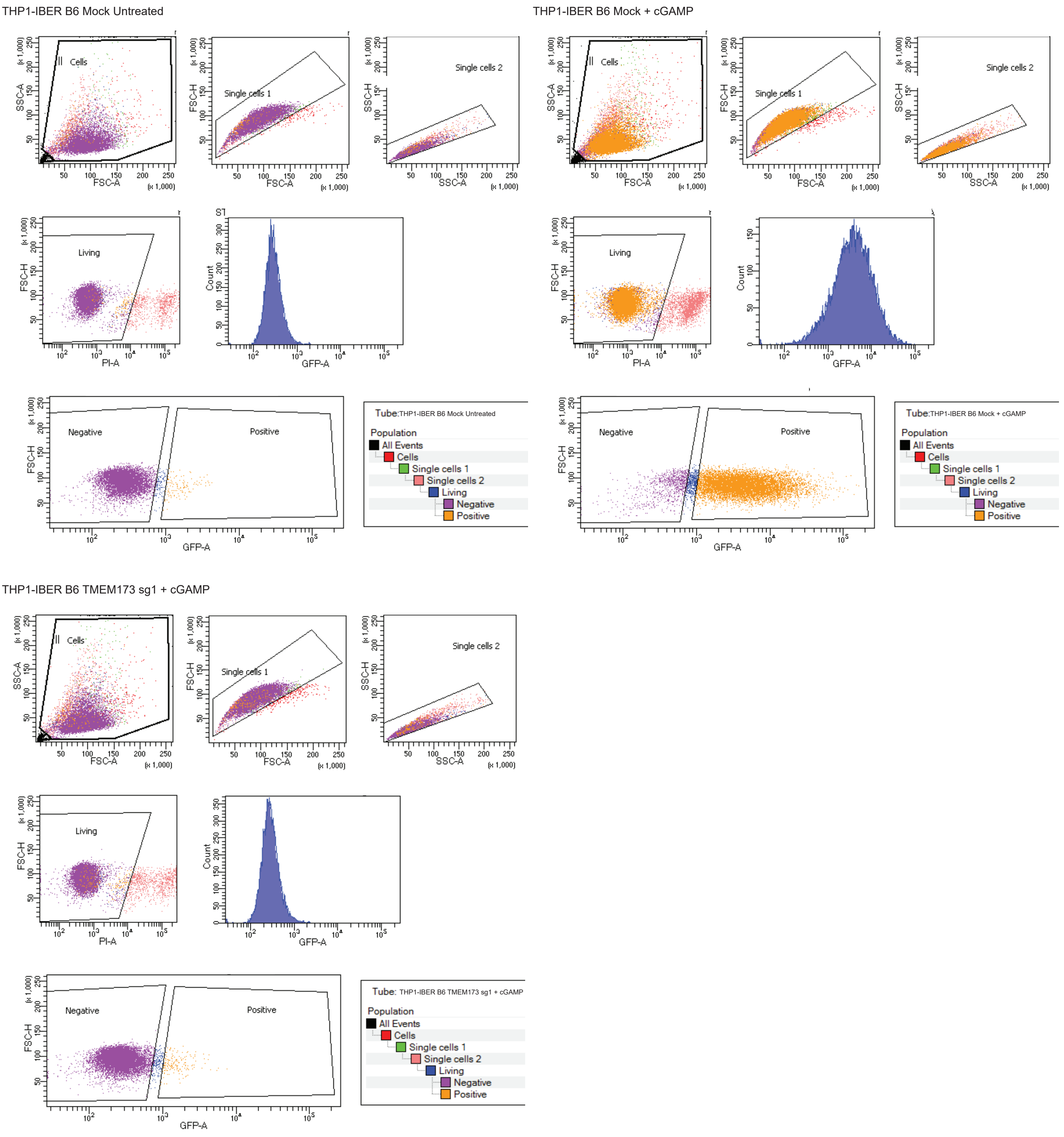

Supplementary Figure S8. Gating strategy used for FACS, 12 hours after treatment with cGAMP (125 µg/mL) THP1-IBER cells were separated based on d2eGFP signal.
